# Clinical Features of Acute Anterior Uveitis with Complicated Course in a Slovenian Patient Population

**DOI:** 10.1155/2020/6079146

**Published:** 2020-09-19

**Authors:** Katarina Vergot, Sasa Pockar, Lan Umek, Natasa Vidovic Valentincic

**Affiliations:** ^1^Eye Hospital, University Clinical Center, Ljubljana, Slovenia; ^2^Faculty of Administration, University of Ljubljana, Ljubljana, Slovenia; ^3^Faculty of Medicine, University of Ljubljana, Ljubljana, Slovenia

## Abstract

**Purpose:**

To report on patients who needed hospitalization due to acute anterior uveitis (AAU) and within this group to compare clinical features and outcomes of treatment of HLA-B27+ and HLA-B27− AAU in the population of Slovenian patients.

**Methods:**

Retrospective study of hospitalized patients with AAU in the last 39 months at the Eye Hospital in Ljubljana. The data of AAU patients were retroactively studied and compared on the basis of HLA-B27 antigen presence: visual acuity upon admission, visual outcome, the presence of hypopyon, fibrinous reaction, posterior iris synechiae, and complications, such as elevated intraocular pressure, cataract, and cystoid macular edema (CME). We compared the investigations in the diagnostic process, the associated systemic disease, and the treatment administered. Statistical analyses included Student's *t*-test Fisher's exact test, and the Kolmogorov–Smirnov test. A *p* value of <0.05 was considered statistically significant.

**Results:**

A total of 37 hospitalized patients with AAU were included. HLA-B27 antigen was detected in 73% of patients. In the HLA-B27+ group, women were more commonly affected, while the males were more affected in the HLA-B27− group. The occurrence of fibrin was significantly more common in HLA-B27+ patients, as well as hypopyon and posterior synechiae; only fibrin reached the statistical significance (*p* < 0.05). The incidence of cataracts, ocular hypertension, and glaucoma did not differ significantly between the two groups. HLA-B27+ AAU was more often associated with systemic diseases, and patients in this group were more frequently treated with systemic immunomodulatory drugs, however, no difference reached the statistical significance. We did not notice any major differences in the final visual acuity in the comparing groups.

**Conclusion:**

Almost ¾ of AAU patients that required hospitalization were HLA-B27+. In this group, disease was more severe, more frequently associated with ocular complications and systemic disease, but final visual acuity was the same in both groups. HLA-B27 typing has no prognostic value in our group of complicated AAU patients, but it eases the decision about necessary diagnostics and treatment.

## 1. Introduction

Acute anterior uveitis (AAU) is the most common form of uveitis and defined as an inflammation of the anterior segment of the uvea with a limited duration of three months. It can occur once or is recurrent. The disease may appear as an isolated one, but it is often associated with inflammation elsewhere in the body [1, 2].

The most common diseases associated with AAU are anterior uveitis associated with the HLA-B27 surface antigen, and many articles describing the linkage of the clinical picture of AAU with the presence or absence of HLA-B27 antigen were published [3–12]. HLA-B27 positivity among AAU patients varies between 12% and 88% [13, 14]. HLA-B27+ AAU is associated with ankylosing spondylitis, psoriatic arthritis, reactive arthritis, and arthritis associated with chronic inflammatory bowel diseases [1, 2, 6, 7, 10, 15, 16].

Patients with AAU mostly do not require hospitalization; however, there are some exceptions. In our paper, we report on clinical characteristics, complications, and treatment of AAU patients who needed hospitalization because of the complicated course of the disease.

## 2. Materials and Methods

We retrospectively reviewed data of 37 consecutive patients with AAU, hospitalized between 1^st^ January 2015 and 31^st^ March 2018 at the Ljubljana Eye Hospital. Hospital treatment was needed because of the disease severity in terms of poor response to outpatient treatment, a prolonged duration of illness, or recurrence of the disease within 3 months after the first episode. The patients were classified into two groups according to the presence of HLA-B27 antigen.

Following features were documented upon admission: gender, age, and best corrected visual acuity (BVCA) with the Snellen chart and intraocular pressure (IOP). Clinical examination was carried out with a slit-lamp. We documented the presence of hypopyon, fibrin formation, posterior iris synechiae, and complications of the inflammation, such as ocular hypertension, secondary glaucoma, cataract, and cystoid macular edema. We noted final BCVA. Optical coherence tomography (OCT) was performed. Other imaging diagnostics (fluorescein angiography, indocyanine green, and ocular ultrasound) were used as needed. Other investigations were indicated according to the clinical picture. X-ray of the sacroiliac joints was performed in all patients with inflammatory back pain or morning stiffness. In patients who had atypical clinical picture, the following investigations were conducted: serology for *Borrelia burgdorferi* and *Treponema pallidum*, QuantiFERON-TB, serum angiotensin converting enzyme level, and chest X-ray. An aqueous humor tap was also performed to exclude viral/bacterial etiology when necessary. The presence of systemic diseases associated with AAU like spondyloarthropathies, inflammatory bowel disease, psoriatic arthritis, or reactive arthritis and investigations needed were documented also.

The study excluded patients who had confirmed viral/bacterial etiology of AAU. For each of our hospitalized patients included in the study, an ophthalmic and general medical history was taken, and the presence of characteristic symptoms and signs typical of uveitis associated systemic diseases were documented. The final diagnosis of systemic disease was performed by a rheumatologist.

Ophthalmic hypertension was defined as an eye pressure higher than 21 mmHg. If there was also an impairment of the optic nerve and the visual field defects, not noted before admission to the hospital, we defined the condition as glaucoma. The types of cataracts resulted from inflammation or treatment and were not specified more precisely. Patients were treated according to the severity of the clinical picture with topical or systemic immunosuppression/immunomodulation.

Statistical analysis was performed using SPSS software. For comparison of two groups of patients (HLA-B27+ and −), different statistical tests were used: the *t*-test for comparison of age (after checking for normality using the Kolmogorov–Smirnov test and Fisher's exact test, for comparison of distribution of gender, Fisher's exact test was also used for statistical analysis of relation between group membership (HLA-B27 +and −) and the presence or absence of systemic diseases, ocular complications, investigations performed in patients, and treatment choices. To measure the strength between group membership and other analyzed properties, odds-ratios (together with 95% confidence intervals) were computed. Due to some 0s in contingency tables, Haldane–Anscombe correction for computation of odds-ratio was used. The level of significance was set at level 0.05. The study followed the tenets in the Declaration of Helsinki.

## 3. Results

### 3.1. General Characteristics

73% of hospitalized patients were HLA-B27+ and all our patients were caucasians. The disease was slightly more common in women in the HLA-B27 +ve group and in males in the HLAB27 −ve group. The age and gender distribution of the patients using median and IQR is listed in the Table 1. Using the Kolmogorov–Smirnoff test, the distribution of age can be determined as normal (asymptotic *p*=0.051). For comparison of both groups, we therefore used the *t*-test. The male to female ratio is lower in the group HLA-B27+ (1 : 1.1) than in HLA-B27− (1.5 : 1). The odds-ratio OR (computed with Haldane–Anscombe correction) equals 0.64, and the 95% confidence interval for OR ranges from 0.16 to 2.65. The association between group membership (HLA-B27+ and HLA-B27−) is not statistically significant (*p*=0.395).

The age of our patients at the first manifestation of the disease did not differ significantly between the two groups in our case (*p* > 0.05), and in both groups, the patients were under the age of 40 years.

In Table 2, we present findings describing clinical features, ocular complications, investigations performed in patients and treatment choices.

### 3.2. Clinical Features

In both groups, HLA-B27+ and HLA-B27−, the disease was more commonly unilateral, 21 out of 27 or 78% and 8 out of 10 or 80%, respectively. 2 patients in the HLA-B27+ group had simultaneous bilateral AAU and 4 had alternating bilateral AAU. In the HLA-B27 group three was 1 patient with simultaneous and 1 with alternating bilateral AAU. From the Table 2, further clinical features are listed: the fibrin exudation in the anterior chamber was significantly more common in HLA-B27+ patients in 17 out of 27 patients or in 63%, while in the HLA-B27− group, we did not detect it (*p*=0.001). This is the only difference between the groups that reached a statistical significance. Posterior synechiae occurred in a slightly higher percentage in HLA-B27+ patients (74%), as compared with the second group (50%). Hypopyon occurred in the HLA-B27+ group only, at 23%.

### 3.3. Complications

All complications listed in Table 2 occurred in both groups, and HLA B27 −ve patients had more complications than HLA B 27 +ve patients, but the difference was not statistically significant.

### 3.4. Investigations

In the process of diagnosis, a number of investigations were conducted in both groups of patients (Table 2). Optical coherence tomography (OCT) was most commonly performed, in 18 out of 27 patients or in 67% of HLA-B27+ and in 7 out of 10 or in 70% of HLA-B27− patients. Other investigations, percentage, and significance in both groups are given in Table 2. There was a distinction in number of other ocular imaging performed between the two groups with more of them performed in the HLAB27− group but did not meet the statistical significance.

### 3.5. Treatment

All patients in both groups were treated with topical corticosteroids and topical mydriatics. Systemic nonsteroidal anti-inflammatory drugs and biological agents were used only in the HLA-B27+ patients group; 26% of HLA-B27+ (7 patients) and 10% of HLA-B27− (1 patient) patients were treated with methotrexate and 11% (3 patients) of HLA-B27+ patients with biologics. Comparison of medication between the two groups, however, revealed no statistically significant differences (*p* > 0.05, Table 2).

### 3.6. Associated Systemic Diseases

56% of our hospitalized HLA-B27+ patients had one or more uveitis-associated systemic diseases, the most common being ankylosing spondylitis, at 41%. One patient had reactive arthritis and one psoriatic arthritis (7.5% of HLA-B27+). In the HLA-B27+ group, there were 7 out of 27 patients known to have systemic disease before the diagnosis of AAU. In 4 out of 27 patients, AAU was the first manifestation of HLA-B27+ associated disease. 3 of them were later diagnosed with ankylosing spondylitis and 1 with reactive arthritis. In the HLA-B27− group, 2 out of 10 patients had other associated systemic illness, one reactive arthritis, and one ulcerative colitis (each encompassing 10% of HLA-B27−). In both of them, the disease was present before the diagnosis of AAU. Ankylosing spondylitis and reactive arthritis were more common in the HLA-B patients group, but the difference with the HLA-B27− group does not meet criteria to be statistically significant (*p* > 0.05).

### 3.7. Best Corrected Visual Acuity at Discharge

The final best corrected visual acuity (BCVA) did not differ between the two groups. Most patients, HLA-B 27+ and HLA-B27−, had a final best corrected visual acuity (BCVA) between 0.5 and 1.0 using the Snellen chart (24 out of 27 or 89% and 9 out of 10 or 90%, respectively). BCVA of less than 0.3 (legal low vision in Slovenia) was noted in 2 patients and was the consequence of complication consequences because of noncompliance to treatment before the admission to the hospital.

## 4. Discussion

Acute anterior uveitis (AAU) is the most common form of uveitis. Although many publications exist on this topic, it is still inadequately understood [1, 2]. In our study, we described clinical characteristics, complications, management, and outcomes of AAU patients with the complicated course of AAU requiring hospitalization. Known AAU association with HLA B27 antigen in Caucasian population has been shown to be around 50% [17]. Our data showed that the most common AAU patient admitted to the hospital was HLA-B27+, in 73%.

In the literature, HLA-B27+ AAU is described as a disease that affects the male population more frequently, [4, 8, 11] in 54–76% [4, 18], and our results showed similarity in HLA-B27-AAU but not in HLA-B27+ AAU.

Although differences were reported regarding the age of onset between the HLA-B27+ AAU and HLA-B27 AAU (between 20 and 40 years) [4, 8, 11] on average, and in HLA-B27− patients, 10 years later according to literature [11, 18]. There was no statistically significant age difference between the two groups of patients, but it was within the previously reported range.

AAU can be associated with inflammation elsewhere in the body [1, 2]. A number of studies have shown a strong association of HLA-B27 antigen associated anterior uveitis with systemic, especially autoimmune diseases, of which ankylosing spondylitis is the most common, which was also confirmed in our study [4, 7, 10, 16, 18, 19]. A significantly greater proportion of HLA-B27+ (more than half) than HLA-B27− patients (less then quarter) had associated systemic disease, which was in accordance with previous studies [4].

Multiple reports have shown that the clinical features of HLA-B27+ and − AAU differ significantly [3, 7, 9, 10, 12, 16, 18]. In our case, the differences between the two groups were also noticeable. Fibrinous exudation and hypopyon were observed in the HLA-B27+ group only, at 63% and 23%, respectively, supporting reported data found in the literature [9–11]. Previous studies showed that posterior synechiae were the most common complication in both groups, and this was similar to data reported by Linsen and Meenken [18].

The clinical course of the disease may be associated with some ocular or systemic complications as a result of inflammation or as a result of treatment. More complications, such as cataract, cystoid macular edema, ocular hypertension, and secondary glaucoma in our study appeared in the HLA-B27− patients group. Some other studies [9] have also described less favourable clinical course and outcomes of HLA-B27+ AAU. On the other hand, Rothova and associates [11] showed greater percentage of eye complications in HLA-B27+ patients. Linsen and Meenken [18] on the contrary did not demonstrate differences in the proportion of complications between HLA-B27+ and HLA-B27− AAU.

It has been reported that patients with HLA-B27+ uveitis usually have more severe clinical picture that is often refractory or less responsive to topical corticosteroid therapy and often requires more aggressive treatment [4, 9, 20, 21]. In our cohort, however, there was only one patient from each group who required systemic steroid. The case was different with systemic immunomodulatory treatment. Some of the patients from both groups required methotrexate to control the recurrences and severity of inflammation, and this was more often necessary in HLA-B27+ patients. In this group, three patients were receiving methotrexate because of their systemic disease (listed in Table 2). Methotrexate is a recognised treatment for the patients with HLA-B27+ AAU where there are multiple vision threatening flares [9], and it was successful in our patients as well. The use of biological medicines in HLA-B27+ AAU is on the rise [3, 4, 9, 22]. Biological agents in our study were used in HLA-B27+ patients exclusively, at 11%. If the treatment was not effective enough, then adalimumab was used—all patients on adalimumab received methotrexate first. Our study confirms that it is more difficult to control HLA-B27+ AAU, which more often required the use of immunomodulatory drugs, although we could not find the statistically significant differences between the groups.

The final visual acuity has already been the subject of numerous studies. The results are not uniform. In most of the studies, the patients reach good final visual acuity [8, 11] and slightly worse final visual acuity in the case of complications involving the posterior segment of the eye [3, 4, 9]. In our study, the final visual acuity did not differ significantly between the two comparing groups, and this outcome was similar to some previous reports [4, 18].

This study offers a valuable insight into AAU with more severe clinical course in a mostly Caucasian population. It clearly shows that the majority of these patients is HLA-B27+. Although this was not a prospective epidemiologic study, our findings suggest that a thorough clinical history and examination is necessary in all patients with AAU, specifically in the light of the fact that it may be the initial manifestation of a spondyloarthropathy, that was the case in 15% of our HLA-B27+ patients and in 26% of the same group that have already had systemic rheumatological disease. The HLA-B27+ patient in our cohort more likely presented with fibrinous exudation than HLA-B27− patient but had less other complications than HLA-B27− patient. The treatment needed in the HLA-B27+ group was more often systemic, although none of the observed parameters reached the statistical significance. Final BCVA between two groups was excellent and the same in both groups.

There are several limitations to this study. It is a retrospective, single centre study with limited number of patients. A larger number would allow better results and better analysis. Also, there is definite bias regarding the study participants due to the referral pattern in this group of patients. Numerous reports have shown that HLA-B27+ patients have more severe disease course and complications, and this might be the reason that most of the hospitalized patients were HLA-B27+. HLA B-27− patients in our cohort were those who had more severe course of disease for various reasons (the most important being previous suboptimal treatment and consequently more complications at the admission already) and do not reflect the HLA-B27− AAU clinical course and complications in the entire population, which is mostly idiopathic and has good outcome with local treatment only. The latter is the reason for higher complication rate in our HLA-B27− AAU group. Moreover, higher frequency of systemic immunomodulatory and biologic treatment in the HLA-B27+ group reflect the actual need for more aggressive treatment approach in this group of patients. We did not analyze the groups throughout the entire clinical course but only at the last presentation at the end of hospitalization; therefore, it is difficult to make conclusive remarks with regards to disease prognosis.

The results show a tendency to associate the presence of HLA-B27 antigen with a more severe form of the disease, since 73% of admitted AAU patients had HLA-B27 antigen. HLA-B27 typing is the first thing to consider in admitted AAU patients, since it directs the extent of investigations, ocular imaging especially, and it directs the need for potentially more aggressive treatment.

## 5. Conclusion

Our study represents a retrospective survey of all complicated AAU patients who need hospitalization at a tertiary uveitis centre at the Eye hospital Ljubljana, with their clinical profile, complications, required investigations, treatment, and outcomes. As it is the only tertiary centre in the country, it offers a valuable overview of complicated AAU cases in one population.

In conclusion, when dealing with an AAU patient with complicated course, there are a few things to consider:

- Even in the AAU with complicated course of the disease, the visual prognosis is good,
- There is a need for strong cooperation with rheumatologists since in HLA-B27+ AAU patients, association with rheumatological disease was strong also in our cohort,
- HLA-B27 typing does not have a prognostic value in our series of patients but importantly leads to necessary diagnostics and decisions about treatment.

## Figures and Tables

**Table 1:** The age and gender distribution of the patients.

	HLA-B27+		HLA-B27−	
	Median	IQR	Median	IQR
Age	35	18,5	36	19,25
Gender	Female 14 (52%)	Male 13 (48%)	Female 4 (40%)	Male 6 (60%)

**Table 2:** Clinical features, ocular complications, investigations performed in patients and treatment choices.

Variable		HLA+		HLA−		Odds ratio	Confidence interval		*p* value
		Percentage	Number (out of 27)	Percentage	Number (out of 10)	With Haldane–Anscombe correction	Upper bound	Lower bound	Using Fisher's exact test
Clinical features	Fibrin exudation	63%	17	0%	0	35.0	1.9	661.0	0.001
	Hypopyon	23%	6 (out of 26)	0%	0	6.7	0.3	129.9	0.157
	Posterior synechiae	74%	20	50%	5	2.7	0.6	11.6	0.240

Ocular complications	Cataract formation	30%	8	40%	4	0.6	0.2	2.7	0.696
	Cystoid macular edema	8%	2 (out of 26)	20%	2	0.4	0.1	2.4	0.353
	Ocular hypertension	15%	4	40%	4	0.3	0.1	1.3	0.174
	Secondary glaucoma	4%	1	10%	1	0.4	0.0	3.9	0.473

Investigations performed in patients	Macular OCT	67%	18	60%	6	1.4	0.3	5.7	0.716
	Aqueous humor tap	30%	8	30%	3	0.9	0.2	4.2	1.000
	Sacroiliac joint X-ray	22%	6	30%	3	0.7	0.1	3.0	0.679
	Serology (Treponema, Borrelia)	26%	7	30%	3	0.8	0.2	3.6	1.000
	QuantiFERON-TB	22%	6	20%	2	1.0	0.2	5.4	1.000
	Aerum angiotensin converting enzyme	4%	1	0%	0	1.2	0.0	31.6	1.000
	Other ocular imaging (FA, ICG, and US)	11%	3	40%	4	0.2	0.0	1.1	0.069

Treatment choices	Topical corticosteroids	1	27	1	10	2.6	0.1	140.7	1.000
	Topical mydriatics	1	27	1	10	2.6	0.1	140.7	1.000
	Periocular corticosteroids	11%	3	10%	1	0.9	0.1	7.1	1.000
	Systemic corticosteroids	4%	1	10%	1	0.4	0.0	3.9	0.473
	Methotrexate	26%	7	10%	1	2.3	0.3	15.2	0.404
	NSAID	4%	1	0%	0	1.2	0.0	31.6	1.000
	Biologics	11%	3	0%	0	3.0	0.1	63.4	0.548

## Data Availability

The data used to support the findings of this study are available from the corresponding author upon request.
